# Pheno-SELEX: Engineering Anti-Metastatic Aptamers through Targeting the Invasive Phenotype Using Systemic Evolution of Ligands by Exponential Enrichment

**DOI:** 10.3390/bioengineering8120212

**Published:** 2021-12-13

**Authors:** Greg Shelley, Jinlu Dai, Jill M. Keller, Evan T. Keller

**Affiliations:** 1 Department of Urology, University of Michigan, Ann Arbor, MI 48109, USA; gregsh@med.umich.edu (G.S.); jldai@med.umich.edu (J.D.); 2 Unit for Laboratory Animal Medicine, University of Michigan, Ann Arbor, MI 48109, USA; jmurtha@umich.edu; 3 Single Cell Spatial Analysis Program, University of Michigan, Ann Arbor, MI 48109, USA; 4 Biointerfaces Institute, University of Michigan, Ann Arbor, MI 48109, USA

**Keywords:** aptamer, prostate cancer, metastasis, invasion, osteosarcoma, murine model

## Abstract

Multiple methods (e.g., small molecules and antibodies) have been engineered to target specific proteins and signaling pathways in cancer. However, many mediators of the cancer phenotype are unknown and the ability to target these phenotypes would help mitigate cancer. Aptamers are small DNA or RNA molecules that are designed for therapeutic use. The design of aptamers to target cancers can be challenging. Accordingly, to engineer functionally anti-metastatic aptamers we used a modification of systemic evolution of ligands by exponential enrichment (SELEX) we call Pheno-SELEX to target a known phenotype of cancer metastasis, i.e., invasion. A highly invasive prostate cancer (PCa) cell line was established and used to identify aptamers that bound to it with high affinity as opposed to a less invasive variant to the cell line. The anti-invasive aptamer (AIA1) was found to inhibit in vitro invasion of the original highly invasive PCa cell line, as well as an additional PCa cell line and an osteosarcoma cell line. AIA1 also inhibited in vivo development of metastasis in both a PCa and osteosarcoma model of metastasis. These results indicate that Pheno-SELEX can be successfully used to identify aptamers without knowledge of underlying molecular targets. This study establishes a new paradigm for the identification of functional aptamers.

## 1. Introduction

Targeting components of the metastatic process may result in preventing the development of metastases or delay the progression of cancer. The metastatic process involves a complex process termed the “metastatic cascade” that involves multiple steps including invasion through the basement membrane, survival in the circulation, extravasation at the distant site, and invasion into the target tissue. Inhibiting any one of these steps should result in preventing metastasis.

Aptamers are small RNA/DNA molecules that are designed for therapeutic or diagnostic use reviewed in [1,2,3]. Aptamers have several advantages over currently used therapeutic molecules. For example, aptamers have greater stability and less immunogenicity than antibodies and aptamers are less technically demanding to produce and less expensive to produce than small molecules. Furthermore, aptamers, when appropriately selected, bind to target molecules with greater affinity than either antibodies or small molecules. Finally, aptamers have been shown to be successful for the treatment of human disease (age-related macular degeneration, a retinal disease) and received FDA approval [4] indicating that they are valid therapeutic modalities; thus, once appropriate aptamers are identified, they could be rapidly translated into clinical trials.

The selection of aptamers for targeting specific isolated molecules was described as systemic evolution of ligands by exponential enrichment (SELEX) reviewed in [5,6,7,8]. Briefly, a library of synthesized aptamers that contain a random sequence of nucleotides to create 10^14^ possible combinations was incubated with a known protein in vitro for positive selection. The protein is precipitated along with any aptamers bound to the protein. The bound aptamers are then isolated and PCR amplified to create a new library and this process is repeated several times to increase the specificity of the final isolated aptamers. Negative selection is also used to delete a non-specific binding. The SELEX method has proven to be a very powerful system to identify specific aptamers that bind known target molecules with great affinity and have led to clinical approval of an aptamer that targets vascular endothelial growth factors for ocular use reviewed in [9]. Aptamers have been demonstrated to target specific proteins in cancer cells, including prostate-specific membrane antigen (PSMA) in PCa cells [10,11]. A modification of SELEX was published that described targeting a specific kinase within cells ex vivo [12]. In all these instances, the aptamer was created by targeting a known protein. Alternatively, in a technique termed Cell-SELEX, intact cells are used to select aptamers that can be used as drug carriers or signal reports [13,14,15]. We hypothesize that subjecting different cells that possess a similar target phenotype to SELEX opens the door to a novel method of creating functional aptamers that target specific cancer cell properties, such as invasiveness. We call the method of using SELEX to target a phenotype Pheno-SELEX. Accordingly, the goal of this study was to use Pheno-SELEX to target a phenotype of cancer cells that is critical to the development of metastasis, namely invasion.

## 2. Materials and Methods

### 2.1. Cell Lines

C4-2B cells (Dr. Leland Chung, Emory University) are derived from LNCaP cells through several passages through nude mice and isolated from bone metastases [16]. PC-3 (ATCC, Manassas, VA, USA) were derived from a human PCa bone metastasis [17]. 82L osteosarcoma cells were derived by serially injecting metastatic tumors from OS187 osteosarcoma cells.

### 2.2. Invasion Assay

In vitro invasion was assessed as previously described with minor modifications [18]. Briefly, the percent in BD BioCoat Matrigel Invasion Chambers (BD Biosciences, Bedford, MA) were used as directed by the manufacturer and as we have previously described [18]. To test the effect of aptamer, 2.5 × 10^4^ cancer cells were seeded in the upper compartments of 24-well transwell chambers for 40 h, with 2–3 μL aptamer at 12 μg/mL added and replaced after 20 h. To determine relative invasiveness, we first determine the invasive cell percentage in the individual cell line (or treatment condition) by dividing the number of cells that invade through the membrane by a total number of cells plated onto the membrane. Then to determine the invasiveness relative to PC-3 Luc cells (or control treatment), we divide the subclone’s percent invasion by PC-3 Luc parental cell line (or control treatment) percent invasion.

### 2.3. Generation of High and Low Invasive PCa Cell Lines

PC-3 Luc cells at 1.25 × 10^5^ cells/mL were added to each well of a six-well BD BioCoat Matrigel Invasion Chamber (BD Biosciences, Bedford, MA, USA) and allowed to invade through the membrane. Invasive and noninvasive cells were isolated by scraping the bottom or top of the membrane, respectively. Then these cells were replated on invasion plates for several cycles as indicated in the results.

### 2.4. Cell Proliferation

Cell proliferation was measured using the CellTiter 96AQ non-radioactive cell proliferation assay (Promega, Madison, WI, USA) as described by the manufacturer.

### 2.5. SELEX Procedures

An RNA library was transcribed as previously described [19], with minor modifications. The DNA template used for transcription was 5′AGTAATACGACTCACTATAGGGAGTCGACCGACCAGAAN_40_TATGTGCGTCTACATCTAGACTCAT. Upon transcription, ribonucleotides with 2′fluro modification on each C and U were used to create a modified library. The resulting RNA was isolated in a 10% PAGE in 8 M urea and extracted by soaking the gel slice overnight at 37 °C in buffer (0.1 M sodium acetate, 1 mM EDTA, and 0.2% SDS) followed by phenol-chloroform and precipitation with ethanol.

PC-3 Luc 5-Inv cells in 100 mm tissue culture plates at 90% confluence were washed 3 times with 10 mL binding buffer (Dulbecco’s PBS with MgCl_2_ and CaCl_2_) and incubated with 100 μL of single-stranded RNA at 0.1 μg/μL (a total of 10 μg of input RNA) and 10 mL binding buffer for 45 min at 37 °C. Seven 10 m washes with 10 mL binding buffer were followed by a final 40 m wash with 5 mL of 10 mM EDTA to detach the cells. Cells were pelleted and the supernatant removed. RNA was isolated using 1 mL Trizol^®^ (Invitrogen, Waltham, MA, USA) and cDNA was prepared from the RNA using Superscript II RNase H reverse transcriptase kit (Invitrogen) with the primer ATGAGTCTAGATGACGCACATA and then amplified by PCR using Taq polymerase with parameters of 94 °C for 5 min, then 30 cycles of 94 °C at 30 s, 50 °C for 30 s, and 72 °C for 30 s, followed by a final incubation of 72 °C for 7 min. The primers used were 3′ N40 primer ATGAGTCTAGATGACGCACATA and 5′ T7 primer AGTAATACGACTCACTATAGGGAGTCGACCGACCAGAA. The PCR product was purified (Qiaquick PCR purification kit, Qiagen, Valencia, CA, USA) and transcribed by T7 RNA polymerase for 4 h at 37 °C using ribonucleotides with 2′fluro modification on each C and U. The aptamers were gel purified as described for the library. After the multiple rounds of selection, the final PCR products from the final round were cloned into GW/TOPO vector (Invitrogen) and positive clones were sequenced. Aptamer secondary structure was determined using CentroidFold (rtools.cbrc.jp/centroidfold; accessed on 5 December 2021) [20].

### 2.6. Animals

8-week-old male nude mice (*nu/nu*) (Charles River, Wilmington, MA, USA) were housed under pathogen-free conditions. The animal protocol was approved by the University of Michigan Animal Care and Use Committee. Intracardiac injection of PCa cells (2.5 × 10^5^ cells of PC-3 Luc in 0.1 mL) was performed as previously described [21]. For tibial tumors, single-cell suspensions (0.5 × 10^6^ cells) of 82L were injected into the right tibia of the male nude mice (*n* = 8/group) as described [22]. Mice were injected daily i.p. with 0.1 mL (360 μg/mL) of 23 mer RNA with 2′fluro modification on each C and U, either aptamer A1A1, AGGGCCUGAGGUAGUGCGCGUUG or scrambled RNA control, GGCGGUACGGCGCUGUGGUUAGA (Dharmacon Inc., Lafayette, CO, USA).

### 2.7. Cellular Fluorescent Imaging

Aptamer binding to the cell surface was determined by labeling RNA with Cy3™ (Label IT^®^ µArray Labeling Kits, Mirus Bio LLC, Madison, WI, USA). Briefly, 5 μg RNA was mixed with 5 μL buffer and 5μL of LabelIT and q.s. to 50 μL with H_2_O, then incubated at 37 °C 1 h and ethanol precipitated then re-suspended in 50 μL H_2_O. PC-3 5-Inv and PC-3 4-Non-Inv cells were seeded in 24 well plates at 4 × 10^4^ cells/well and 5 μL labeled RNA added to each well. This was incubated at 37 °C 1 h and washed 3 times with PBS then fixed in 4% paraformaldehyde solution. The cells were imaged on a fluorescent microscope.

### 2.8. In Vivo Bioluminescent Imaging

Tumor burden in vivo was analyzed using bioluminescent imaging (BLI) (Xenogen, Caliper Life Sciences, Hopkinton, MA, USA) as previously described [23]. Photon counts were quantified using Living Image software version 3.2.2 (Caliper Life Sciences).

### 2.9. Detection of Circulating Tumor Cells

Identification of circulating human tumor cells was performed as described [24]. Whole blood was subjected to genomic DNA extraction (Qiagen). Real-time PCR (QPCR) was performed using 15.0 µL of TaqMan PCR Master Mix (Applied Biosystems, Foster City, CA, USA) with 100 nM of human Alu TaqMan probes (F—5′-CAT GGT GAA ACC CCG TCT CTA-3′, R—5′-GCC TCA GCC TCC CGA GTA G-3′, and TaqMan probe—5′-FAM-ATT AGC CGG GCG TGG TGG CG-TAMRA-3′) and 1 µg of the isolated genomic DNA in a total volume of 30 µL. Cycle conditions were 50 °C for 2 min, 95 °C for 10 min followed by 40 cycles of 95 °C for 15 s, and 60 °C for 1 min. Expression was detected as an increase in fluorescence using a sequence detection system (ABI PRISM 7700; Applied Biosystems, Waltham, MA, USA). The DNA levels were expressed as relative copies (% control) normalized against total DNA concentration. Numerical data were determined against a standard curve established using murine cells containing log-fold dilutions of human tumor cells.

### 2.10. Statistical Analysis

Data were described using mean values and standard deviation (SD). Univariate comparisons were made using Student’s *t*-test and multivariate comparisons were assessed using One Way ANOVA and Fisher’s probable least significant difference for post-hoc analysis. Statistical significance was determined at *p* < 0.05.

## 3. Results

### 3.1. Creation of High and Low Invasive PCa Cell Lines

**Figure 1:**
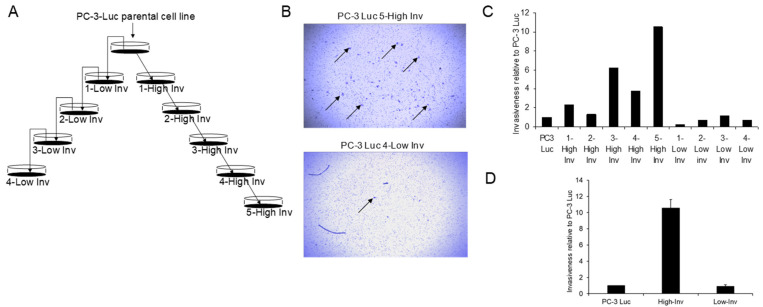
Development of invasive and non-invasive lines of PC-3 Luc. (**A**) Schema of selection of high and low invasive PC-3-luc cell lines. PC-3-Luc cells were placed in a BioCoat Matrigel Invasion Chamber. After 12 h, cells that had invaded through the membrane or stayed on top of the membrane were isolated to create high or and low invasive cells, respectively. This process was repeated as indicated. (**B**) The lower membrane was stained to demonstrate the in vitro invasiveness of invasive cells. Arrows, PC-3 Luc cells. (**C**) Relative invasiveness of successive generations of PC3-Luc lines selected on their ability to invade compared to parental PC-3 cells. (**D**) Invasiveness of final selected high and low invasive cells relative to the parental PC-3 Luc cells (measured in triplicate) Data shown as mean ± SD.

To identify aptamers that have enhanced binding to invasive cells we created PC3 cell lines with a highly invasive capability to be used for positive selection and a low invasive capability to be used for negative selection. To accomplish this, we subjected PC-3 cells to a series of modified Boyden assays as depicted in Figure 1A. Briefly, PC-3 cells were placed on the collagen gel of the modified Boyden chamber. To obtain highly invasive cells, the cells that migrated through the membrane were harvested and then used for the next round of selection for five rounds of selection. In contrast, to obtain low invasive cells, the cells that did not migrate through (i.e., were still on top of the gel), were harvested and used for the next round of selection for four rounds of selection. Using this method, we developed a clone of cells that had a 10.5-fold increase of invasive ability compared to the parental PC-3 cells (Figure 1B–D). In contrast, there was only a limited (approximately 23%) decrease in the invasive ability in the cells selected for low invasion. However, these results demonstrate that we had obtained high and low invasive cells for aptamer selection.

### 3.2. Selection of Anti-Invasive Aptamers

**Figure 2:**
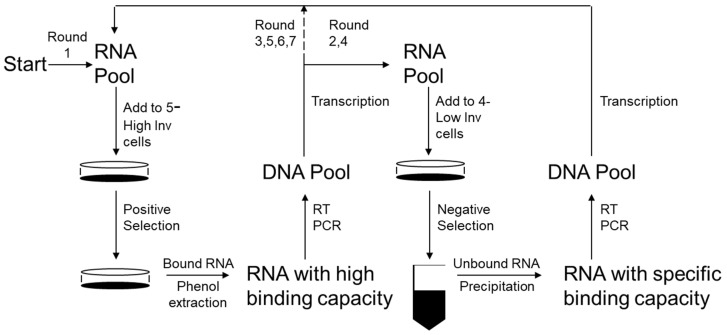
Schematic of SELEX procedure. RNA with high binding capacity was obtained from an RNA pool of 10^14^ RNA molecules 103 bp long containing a 40 bp random sequence flanked by two primer binding sites by adding to highly invasive cells and extracting the bound RNA, which was used to prepare an RNA pool with specific properties. The RNA with general binding capabilities was then removed by mixing the pool with noninvasive cells and using the unbound RNA to prepare a fresh RNA pool.

**Figure 3:**
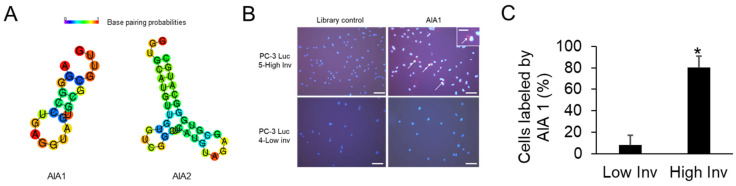
Binding of aptamer A1A1 to invasive and non-invasive PC-3 Luc cells. High invasive and low invasive cells were incubated with fluorescently labeled aptamer A1A1 or RNA pool that had not been subject to SELEX procedure. (**A**) Predicted secondary structures of anti-invasive aptamers. (**B**) Fluorescent images of cells incubated with aptamers. The red color indicates the presence of bound RNA. Arrow, Aptamer bound to surface of a cell. Inset, Cell with bound aptamer. Bar in main figures = 50 μm; bar in inset = 20 μm. (**C**) The percent of cells bound by AIA1 were determined in low and high invasive images from two independent samples. Data are shown as mean ± SD. * *p* < 0.05.

Using the high and low invasive PC-3 cells, SELEX was performed as previously described [12] with modifications to identify a function of cells as detailed below and in Figure 2. Fluoro-modified RNA nucleotides were used as they are resistant to RNases and have greater stability in vivo. Positive selection using the RNA aptamer library pool of 10^14^ aptamers and highly invasive PC-3 cells was performed first. Then counter selection, using low invasive PC-3 cells to eliminate the aptamers that bind the variety of cell surface proteins present on PCa cells that are not associated with the increased invasive phenotype, was performed. This was then followed by a round of positive selection to further amplify the appropriate aptamers, followed by negative selection with parental PC-3-luc cells to again eliminate any aptamers binding non-invasive proteins or low levels of invasive proteins. Finally, this was followed by three rounds of positive selection. The remaining aptamer pool was then cloned into a vector and 20 clones were randomly selected and sequenced. Sixteen clones encoded unique sequences (Table A1); however, two clones each encoded sequences that were represented twice, anti-invasion aptamers 1 and 2 (AIA1: AGGGCCUGAGGUAGUGCGCGUUG and AIA2: GGUGCAUGUUGUGTCGGCUUCAUGUAGAGCGUGGGCAUGC), which suggested that they were good candidates for further exploration (Figure 3A). To ensure the aptamers had enhanced specificity for binding to highly invasive cells compared to low invasive cells we tagged the aptamer with a fluorescent probe and examined its binding ability on the cells. AIA1 is selectively bound to highly invasive cells compared to low invasive cells (Figure 3B,C) indicating that the selection process was successful.

**Figure 4:**
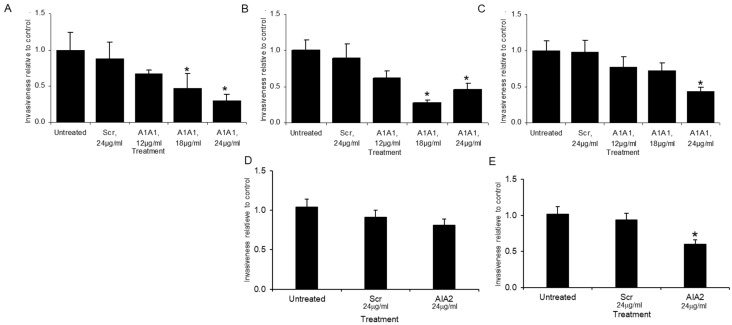
Invasiveness of cancer cell lines after incubation with anti-invasive aptamers or control aptamer (Scr). (**A**–**C**) Cells were incubated untreated or with either 24 μg/mL control scrambled aptamer (Scr) or three concentrations of AIA1 at 12, 18, and 24 μg/mL and the proportion of cells passing through a membrane relative to untreated cells was determined. (**A**) PC-3 Luc 5-Inv cells. (**B**) C4-2B prostate cancer cells. (**C**) 82L osteosarcoma cells. (**D**,**E**) Cells were incubated untreated or with either 24 μg/mL control scrambled aptamer (Scr) or AIA2 at 24 μg/mL and the proportion of cells passing through the membrane relative to the untreated cells was determined. (**D**) PC-3 Luc 5-Inv cells. (**E**) C4-2B prostate cancer cells. Results are reported as mean ± SD of three individual experiments. * *p* < 0.05 versus scrambled control.

Initial evaluation of aptamers efficacy on tumor cell invasion. We next determined the effects the aptamers had at the cellular level. We first evaluated the aptamers’ impact on in vitro proliferation of PC-3-luc cells. The aptamers AIA1 and AIA2 (or scrambled controls) at 0, 1, 2.5, 5, and 10 μg/mL had no effect on proliferation over a 72-h period (not shown). We next evaluated the ability of AIA1 to inhibit invasion of the highly invasive PC-3-luc cells which were the cells used to select the aptamers. Cells were plated in media containing no aptamer, mixed aptamer control (at the high level of test aptamer concentration), or at increasing levels of AIA1. Cells were incubated over 40 h with replacement of aptamers at 20 h, and then invasion was quantified. Increasing levels of AIA inhibited invasion by up to 70% of the level of invasion observed in the control aptamer cultures (Figure 4A). To determine if these results were unique to PC-3 cells or possibly applicable to PCa in general, we tested the impact of AIA1 on another PCa cell line, C4-2B, which is unrelated to PC-3, and found similar results (Figure 4B). Finally, to determine if the effect of AIA was specific to PCa or perhaps applicable to cancers of other tissue origin, we tested the ability of AIA1 against a non-PCa cell line; specifically, we used a highly metastatic osteosarcoma cell line, 82l. AIA1 inhibited invasion of 82l by 60% (Figure 4C). We additionally assessed the ability of AIA2 to inhibit invasion of the highly invasive PC-3 luc cells and C4-2B cells. AIA2 inhibited invasion of C4-2B but not the invasive PC-3 luc cells (Figure 4D,E). Taken together, these results suggest that AIA1 can inhibit in vitro invasion non-cell-or tissue-specific fashion and may be more effective than AIA2.

Finally, to ensure that the SELEX selection process could be reliably used to achieve the identification of aptamers that could inhibit invasion, we repeated the entire SELEX process using unmodified RNA aptamers. The selection process was identical to the above and we identified two aptamers (of different sequence than AIA1 and AIA2) that also effectively inhibited in vitro invasion of the cancer cells. These results indicate that pheno-SELEX is reliable. For further studies, we continued using the modified aptamers as they would be more applicable to clinical utilization.

### 3.3. AIA1 Inhibits Metastasis In Vivo in a Murine Model

**Figure 5:**
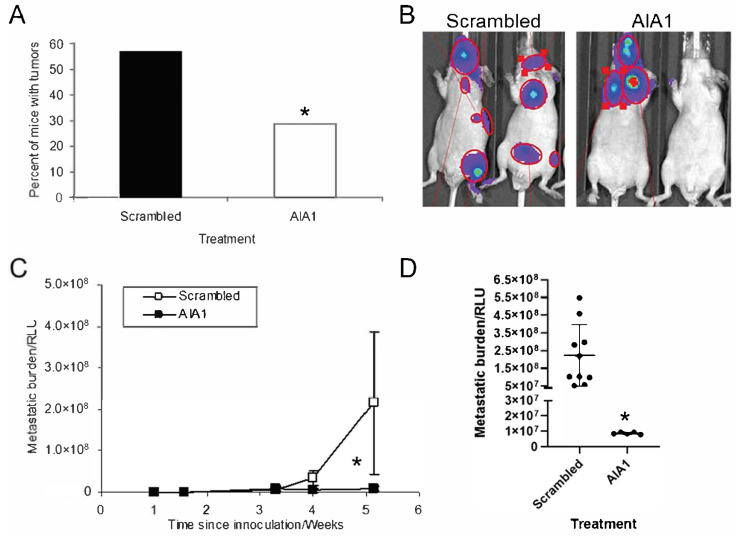
Tumor burden in nude mice following intracardiac injection of PC-3 Luc 5-2 inv cells. 2.5 × 10^5^ cells of PC-3 Luc in 0.1 mL were injected into the left ventricle of the heart (*n* = 20 mice per treatment group) and treated with scrambled control or AIA1 aptamer daily by I.P. injection for seven days. Mice were subjected to bioluminescence imaging (BLI) weekly to determine tumor burden. (**A**) Percent of mice that developed metastases. * *p* < 0.05. *(***B**) Demonstration of BLI. Circled lit areas indicate tumor growth and were used as regions of interest for the calculation of tumor burden. (**C**) Tumor burden over time-based on BLI. Results are reported as mean ± SD. * *p* < 0.05. (**D**) Tumor burden at day 36 post-tumor inoculation based on BLI. Individual animals’ RLU expression are indicated. (Note, one Scrambled and one AIA-treated mouse died on days 8 and 10, respectively, due to peritonitis and were not counted in the tumor burden results). Results are reported as mean ± SD. * *p* < 0.05.

Although in vitro invasion generally correlates with in vivo metastatic ability it does not always reflect in vivo metastatic potential. Accordingly, we next determined if AIA1 could inhibit metastasis of PC-3 cells in vivo. For the initial approach, we used cardiac injection into the left ventricle. This model only recapitulates the latter stages of metastasis (i.e., survival in blood, extravasation, and seeding of cancer cells, invasion into target stroma, and metastatic tumor growth). Thus, there are some weaknesses in the model, but we felt it would provide an initial proof of concept to determine if AIA1 would impact the later stages of metastasis. To accomplish this, PC-3-luc cells were injected via the intracardiac method [21]. Administration of AIA1 or scrambled control aptamers was initiated at the time of tumor cell injection. AIA1 or scrambled control aptamers were administered daily by I.P. injection for 7 days. Bioluminescent imaging (BLI) was performed approximately weekly. Aptamer dose was based on an estimate to achieve blood levels similar to the effective in vitro levels (24 μg/mL) and estimating a mouse blood volume of approximately 3 mL: i.e., 24 μg/mL × 3 mL/mouse = 72 μg/mouse per day). AIA1 administration reduced the number of mice that developed distant tumors by 50% (Figure 5A). AIA administration inhibited total metastatic burden, as determined by BLI, by ~70% compared to the scrambled control aptamer at 4 weeks (Figure 5B–D). Although one mouse from each group died from peritonitis prior to day 11 of treatment, the mice had no toxicities associated with aptamers that we could observe. These results demonstrate several important concepts: (1) AIA1 has an inhibitory effect on the development of metastatic burden in vivo; (2) our selected dosage was effective, and (3) the short-term administration of aptamer had an effect that impacted metastatic growth several weeks after its administration. Additionally, these data suggest that there may be an anti-proliferative vs. pure anti-metastatic effect, as the tumors still grow at distant sites, but their growth is delayed. This effect may be very important clinically as it could potentially delay the progression of established metastases.

### 3.4. AIA1 Inhibits Development of Metastases in Osteosarcoma, a Non-Prostate Tumor

**Figure 6:**
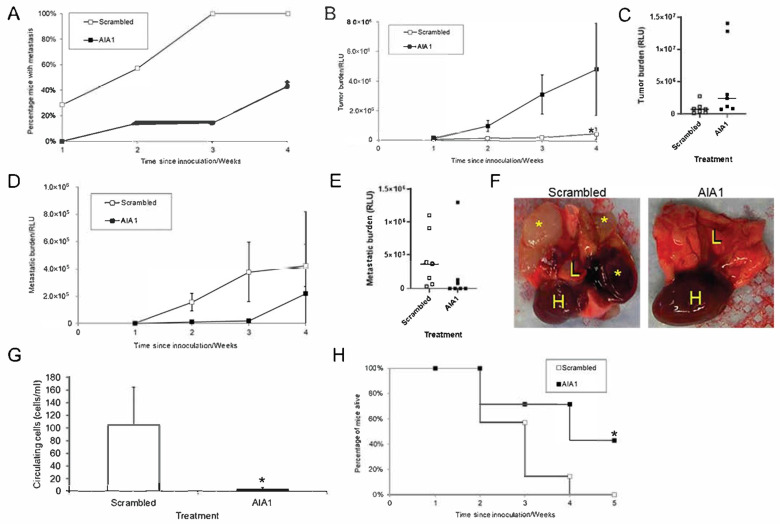
Effect of anti-invasive aptamer AIA1 in a murine model of metastatic osteosarcoma. Osteosarcoma cells (82L cell line) were injected into the right tibiae of male nude mice (5 × 10^5^ cells per injection). At the time of tumor injection, AIA1 or control scrambled aptamers were initiated IP daily for 7 days. Primary and lung metastatic tumor burden was measured using bioluminescent imaging (BLI) weekly. (**A**) Percent of mice with BLI-detectable tumors over time. * *p* < 0.05. (**B**) Total tumor burden per group as measured using BLI. * *p* < 0.05. (**C**) Tumor burden at week 4 post-tumor inoculation based on BLI. Individual animals’ RLU expression are indicated. (**D**) Total metastatic tumor burden per group as measured using BLI. (**E**) Total metastatic burden at week 4 post-tumor inoculation based on BLI. Individual animals’ RLU expression are indicated. (**F**) Lung and heart of mice that had orthotopic implants of 82L and received scrambled aptamer or AIA1. H = heart; L = lung; * = macrometastasis. Note that in the treatment with a scrambled control aptamer the lung tissue is replaced by metastatic nodules some of which are tan and another is hemorrhagic (dark red). In the AIA1-treated group, the lung appears normal. (**G**) Whole blood was obtained at week 1 post-inoculation of tumor at the time of the last aptamer injection to identify circulating tumor cells using a PCR assay. Circulating cells/mL of blood are reported. * *p* < 0.05. (**H**) Survival following intratibial injection with tumor cells. All results were reported as mean ± SD. * *p* < 0.05.

As the SELEX procedure was performed on a PCa cell line, it was plausible that the aptamers would be effective against only PCa cells in vivo. However, since we targeted the phenotype of invasion, it was also possible that the aptamer targeted a general function of invasion that was relevant to multiple cancer types. To determine if AIA1 was specific to PCa or had a more general effect we determined if AIA1 would impact the development of spontaneous metastasis in a model of primary osteosarcoma (OSA). OSA develops in bone and has a high metastatic rate to the lung. To recapitulate the pathobiology of OSA metastasis, we use a model that consists of injecting the metastatic 82L human OSA cell line into the tibia, which is an orthotopic site of tumor growth and thus replicates primary OSA. These tumors readily metastasize to the lung, thus replicating the pathobiology of OSA. Thus, this model is highly effective at recapitulating the entire metastatic cascade. 82L cells were injected into the tibiae of mice and AIA1 or scrambled control aptamers treatment was initiated. AIA1 was administered for a total of 7 days. At all time points, the administration of AIA1 resulted in a decreased number of mice with metastases (Figure 6A). At 4 weeks, when the study was terminated, the AIA1-treated group had a metastatic incidence of 40% as opposed to 100% in the scrambled control aptamer-treated group (Figure 6A). Primary tumor growth was similar between the scrambled control and AIA-treatment groups (Figure 6B); however, there were two mice in the AIA1-treated group that had larger tumors compared to all the other mice which resulted in a large variance in the primary tumor measurements (Figure 6C). In contrast, starting at 2 weeks post-inoculation of tumor into tibiae, mice receiving the AIA1 had a marked decrease in lung metastatic burden compared to those receiving scrambled control (Figure 6D–G). However, at 4 weeks, the total metastatic burden between the treatment groups was similar. It appeared this was due to one extremely large metastasis in the AIA1 treatment group (Figure 6E). While there is no statistical difference when all mice are considered, removal of the mouse with the very large outlier from the statistical analysis results in a *p*-value of 0.03 between the scramble and AIA1 groups. To determine if AIA1 inhibited the development of metastases at the metastatic site or earlier in the metastatic cascade, we quantified the number of circulating tumor cells. Administration of AIA1 was associated with a >90% decrease of circulating tumor cells compared to administration of scrambled control aptamer (Figure 6G). This corresponded with the lower metastatic burden in the AIA1 treatment group. The median survival with mice treated with scrambled aptamer was 21 days; whereas, the median survival of mice that received AIA1 was 28 days, with over 40% of the mice alive at the end of the study compared to 0% in the scrambled aptamer treatment group (Figure 6H). These results indicate that AIA1 specifically inhibits the development of metastasis from a primary tumor, as opposed to having a general anti-tumor growth effect. Furthermore, that the AIAs work on OSA, in addition to PCa, indicate that AIA1 targets a general pro-metastatic feature of cancer cells.

## 4. Discussion

Successfully targeting the development or progression of metastasis would provide great gains for cancer therapy of patients. Towards that end, we initially created a highly invasive PCa cell line that allowed us to use a novel application of SELEX, we term Pheno-SELEX, to successfully develop aptamers that target a critical step of the metastatic process. This method moves away from the paradigm of targeting a known molecule or signaling pathway and offers an innovative approach to targeting cellular functions that are critical for cancer progression. The observation that primary tumor growth is not impacted; whereas, metastasis was inhibited supports the concept that the AIA1 specifically inhibits a metastatic event. Additionally, that AIA1 was effective against a tumor of non-prostate origin indicates that this strategy targeted a general function of metastatic cells.

Initially, SELEX was used to target known molecules in vitro [5]. It has been shown to be highly successful at identifying aptamers with a high affinity for specific target molecules. Ongoing development of SELEX lead to its use to target known molecules ex vivo [13,15]; i.e., in cells transfected with the target molecule such as RET receptor kinase, which is a challenge to target using currently available therapies due to it being a large transmembrane molecule [12]. Advances in SELEX include the use of nanoparticle-mediated strategies which can increase the efficiency of aptamer generation [25]. These methods are efficient strategies for the creation of specific aptamers. However, in many cases, such as cancer metastasis, there are a large number of molecules that mediate the ultimate phenotype. In the case of metastasis, many molecules have been identified that are suggested to promote invasion and metastasis [26,27,28,29]. However, multi-component complexes that may form at the cell surface and their relative importance in the context of other pro-metastatic molecules are unknown. In fact, when some of these proteins bind to other proteins, their conformation may change creating novel epitopes. Accordingly, we reasoned that using SELEX, we could target a phenotype of interest, without knowledge of the underlying molecules or epitopes that mediate the phenotype. We elected to target invasion as it is a key function for the development of metastasis. Based on our data, the strategy was effective.

Our observation that selection of an aptamer through the use of a PCa cell line resulted in the production of an aptamer that was also effective against a different tumor type (i.e., osteosarcoma) provides evidence that the aptamer was targeting a general metastatic phenotype. This supports that a major strength of this method is that it is not critical for the efficacy of the aptamer to know ahead of time or identify what molecule(s) the aptamer targets. This could bring real gains to clinical therapy of metastasis as we can target phenotypes without a priori knowledge of the proteins that mediate metastasis.

One question that remains is what does the aptamer bind? It could be very challenging to identify what the aptamer binds as the aptamer may be targeting an individual molecule or a novel epitope created by a complex of molecules. In the future, this could be explored using affinity chromatography and mass spectroscopy with aptamers as the selected material. Additionally, in silico approaches may be used to identify candidate targets that aptamers can bind [30]. However, a key concept and strength of this methodology are that is not necessary to know what structure the aptamer binds for it to be an effective therapeutic molecule.

There are multiple potential applications of the Pheno-SELEX methodology. For example, identification of aptamers that bind cancer versus non-cancer cells could be used for imaging by the construction of aptamers that are bound to fluorescent signaling molecules [31,32]. For example, SELEX was used to identify aptamers that preferentially bind to small-cell lung cancer cells as opposed to non-cancer cells to be used as prognostic molecules [33]. Aptamers could be designed to specifically target metastatic cells for delivery of radionucleotides [34]. In addition to the already approved use of aptamers for macular degeneration [4], advances in the clinical use of aptamers suggest that they will become viable therapeutic tools in the next few years [35,36,37]. Thus, the identification of aptamers that functionally inhibit cancer phenotypes can be poised for clinical use.

In summary, we have demonstrated a method to target a cancer phenotype without knowledge of the underlying molecules that mediate the phenotype. This approach could lead to rapid advances in cancer therapy, particularly as mechanisms of cancer are many times elusive. Thus, this method could offer some advantages over small molecule inhibitors of cancer for which a mechanistic understanding of what molecules are being targeted is needed.

## Data Availability

Data are available within the article and Appendix A Table A1. Any data not included in the article are available upon request from the corresponding author.

## Appendix A

**Table A1:** Sequences identified in aptamer screening. Mod 1 and Mod 13 sequence was repeated twice (sequence was renamed AIA1) and Mod 10 and Mod 12 sequence was repeated twice (sequence was renamed AIA2); whereas other aptamers were identified once. Length indicates the sequence of the random sequence that was identified in the aptamer. N indicates the number of bases that could not be clearly sequenced.

Clone	Sequence	Length	N
mod10	GGTGCATGTTGTGTCGGCTTCATGTAGAGCGTGGGCATGC	40	0
mod12	GGTGCATGTTGTGTCGGCTTCATGTAGAGCGTGGGCATGC	40	0
mod1	AGGGCCTGAGGTAGTGCGCGTTG	23	0
mod13	AGGGCCTGAGGTAGTGCGCGTTG	23	0
mod6	TATTCGTGTCGAAGGCAGCCTCTTGCATATTTATTGGCGG	40	0
mod8	GCTTATCACCAGCTGTTAGTCATATGCCGTGTGTTGGCC	39	0
mod3	TAGCTGTCCTAACACGGTAATAGTACGCTTATGCTGCCCG	40	0
mod9	TTCAGCGACCGCACGGT	18	0
mod5	AGGTTAGGGCTGAAGCTATTCCTTAGTACCGTGGCCTCTGG	41	0
mod7	GTGTACGGCTATTCCATTCTCCCTGTGGACATGTGCGGCG	40	0
mod2	CAACATAATGTGGCGTATCGACGTTTTAGGTTGTGGACCG	40	0
mod18	CTAGCACACTGACACTGCACGAGTGGGCTGCNCCGCTGGC	41	0
mod15	GCNCNTGGNCTNAGTGCANGNTNCAGGGATGCCGCGNGCG	40	7
mod19	GTTGCGTTGTGCTTGCTGTGTCGCAGAACAGGCGGGAAGG	40	0
mod16	CCATTCATTAGTGTGCCTCCCCTTCTGCAATGGTTGTGGC	40	0
mod20	CGACACGGTACATTGGGTTGGCTTACTTGTGAGTGGGCGG	40	0
mod14	AGCGNGNTGTGCCGAGCNGCGNNGCCANGGTNTGGCGGCC	40	7
mod17	CGCACANNGTGTNANCGCCATGNGGGCCTTGGNGGCGCGG	40	6
mod11	CCATGTTTGATAAGTCACGGGGTCGCAAACGTAGGGGCCA	40	0
mod12	GGTGCATGTTGTGTCGGCTTCATGTAGAGCGTGGGCATGC	40	0
